# Pseudo-Outbreak of *Bordetella parapertussis* Caused by Contaminated Swabs in the Netherlands

**DOI:** 10.3201/eid2804.212097

**Published:** 2022-04

**Authors:** Jacky Flipse, Angelino T. Tromp, Janneke Bosman, Christine ten Hove, Hans Beks, Titia Kortbeek, Guido J.H. Bastiaens, Ellen M. Mascini

**Affiliations:** Rijnstate, Arnhem, the Netherlands (J. Flipse, A.T. Tromp, J. Bosman, C. ten Hove, G.J.H. Bastiaens, E.M. Mascini);; Safety and Public Health Services Gelderland Midden, Arnhem (H. Beks);; National Institute for Public Health and the Environment, Bilthoven, the Netherlands (T. Kortbeek)

**Keywords:** Bordetella, Bacteria, respiratory infections, Bordetella parapertussis, healthcare-associated infections, the Netherlands

## Abstract

An increase in positive *Bordetella parapertussis* tests among patients in a teaching hospital in the Netherlands resulted in enhanced infection control and microbiological surveillance. Further analysis revealed that batches of contaminated nasopharyngeal swabs were associated with a pseudo-outbreak, resulting in incorrect diagnoses, antimicrobial treatments, isolation precautions, and public health notifications.

We report a pseudo-outbreak of *Bordetella parapertussis* in the Department of Pediatrics in Rijnstate, an 809-bed teaching hospital in the Netherlands. The department provides level II care to infants, neonates, and preterm infants. In March 2021, we diagnosed *B. parapertussis* in 3 infants hospitalized for respiratory symptoms by using an in-house PCR against insertion sequences (IS) *IS481* and *IS1001* (*1*). During calendar week 21 (Figure), we identified more *B. parapertussis* cases in the same department, bringing the total case count to 5 in neonates, 1 in a toddler, and 6 in infants. Several of these patients were born prematurely.

**Figure:**
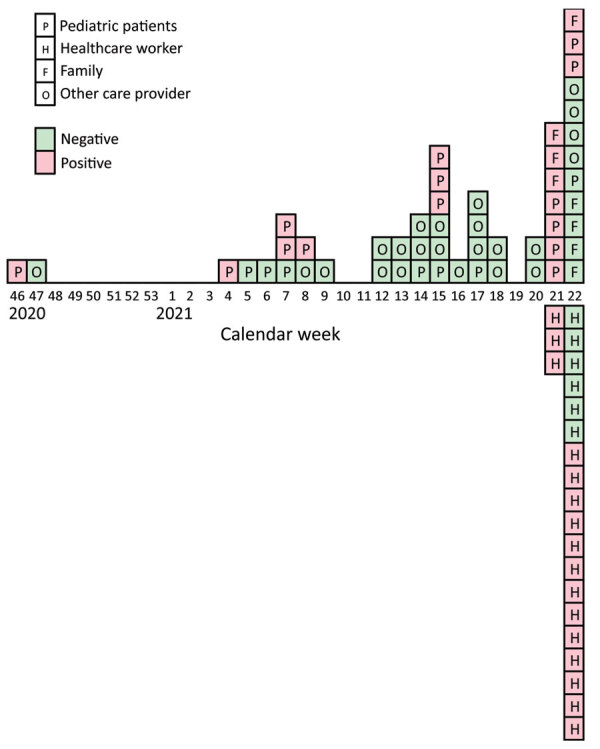
Epidemiologic timeline of *Bordetella parapertussis*–positive findings in a pseudo-outbreak caused by contaminated swabs, the Netherlands, 2020–2021. PCR results positive and negative for *B. parapertussis* are denoted for pediatric patients, their family members, healthcare workers, and other care providers.

PCR-positive case-patients had pertussis-like complaints, and we confirmed *B. parapertussis* in the patients or their siblings. We traced all positive tests to the Department of Pediatrics. Because we suspected nosocomial transmission, we started contact tracing investigations among parents and healthcare workers (HCWs) and identified *B. parapertussis* in another 4 patients and in 3 HCWs. 

Cases among HCWs were particularly unexpected. Because of the coronavirus disease (COVID-19) pandemic, all HCW were using type IIR surgical masks and keeping >1.5 m distance from each other. In addition, all patients had private rooms, and we observed no increase in other respiratory pathogens.

Because we discovered additional *B. parapertussis*–positive cases, we upgraded HCW masks to FFP1, the recommended type for pertussis (*2*). We also confirmed instructions regarding continued HCW social distancing, including during lunch breaks, to prevent further *B. parapertussis* spread. We then implemented extended screening for asymptomatic cases among all HCWs and relatives of *B. parapertussis*–positive case-patients. Among 22 HCWs tested, 72% (16/22) tested *IS1001*-positive.

Parallel to actions in the clinic, we checked the possibility of laboratory contamination. The laboratory uses several controls to confirm sensitivity and specificity of assays; all controls consistently showed correct results. Swipe-tests did not reveal contaminated surfaces or equipment. All cases had relatively high cycle threshold (C_t_) values (median C_t_ 35), as seen with prior *B. parapertussis* results from the laboratory (n = 17). In addition, 2 other laboratories confirmed *B. parapertussis* in original clinical samples and DNA eluates by targeting diverse regions of the *IS1001* gene using an in-house PCR (*1*) or Real Accurate Quadruplex Bordetella PCR (PathoFinder, https://www.pathofinder.com). Confirmatory PCR tests also had high C_t_ values.

Finally, we tested unused ESwab 483CE nasopharyngeal swabs (Copan, https://www.copanusa.com), which included a flocked swab and 1 mL of liquid Amies medium in a plastic, screw cap tube. Liquid Amies media from 7 batches (1 sample per batch) and flocked tips from 2 batches (2 tips per batch) were available for testing. All liquid Amies media were PCR-negative, but both batches of flocked swab tips were PCR-positive for *IS1001*. Moreover, 2 flocked tips were placed in 0.5 mL of Milli-Q water (Millipore, https://www.emdmillipore.com), a 4-fold higher concentration than for standard diagnostic tests. In the higher concentration, we saw lower C_t_ values (C_t_ ≈35) compared with regular diagnostic tests (C_t_ >37).

We retested all 23 PCR-positive HCWs by using individually packaged 503CS01 flocked swabs (Copan) from a PCR-negative batch; 22 HCWs tested PCR-negative and 1 tested PCR-positive. Upon re-examination, we found that testing for the positive HCW case was not performed with an individually packaged swab provided by the laboratory but an ESwab from the suspect batch. Although unintentional, this case proved that the *B. parapertussis* could be traced to *IS1001*-positive nasopharyngeal swabs tips. No *B. parapertussis* could be cultured, which aligns with the notion that the swabs are gamma-irradiated after packaging. Gamma irradiation kills bacteria but does not affect DNA. 

We alerted clinical and molecular microbiologists in the Netherlands, the supplier, and the Health Inspectorate regarding swabs contaminated with *B. parapertussis* *IS1001*-containing DNA. Subsequently, >6 laboratories in the Netherlands recognized and reported false-positive *B. parapertussis* to the National Institute of Public Health and the Environment. Contamination appeared to be associated with specific ESwab batch numbers (Appendix), which explains why the pseudo-outbreak focused on 1 department in our hospital. The contamination was confirmed by the manufacturer, but the source was not disclosed.

*B. parapertussis* can cause pertussis-like symptoms, although symptoms usually are milder and occur less frequently than with *B. pertussis* (*3*). Each year, ≈6,400 *B. pertussis* cases, are notified in the Netherlands based on culture, PCR, or serology, but only 26 *B. parapertussis* cases are notified (Appendix Figure). During 2020–2021, the COVID-19 pandemic and associated social distancing measures caused a large decrease in reported *B. pertussis* cases. Of note, *B. parapertussis* reports did not diminish during this period (Appendix), possibly because testing strategies changed, contaminated swabs were already circulating, or both.

Our report illustrates the importance of critically evaluating of microbiological results lacking clinical and epidemiologic clues. We were confronted with a growing number of neonatal patients and HCWs with unexplained *B. parapertussis*–positive tests. Because these tests are requested infrequently, it took months before contamination with *IS1001*-like DNA in nasopharyngeal swabs became clear. Clinicians and public health agencies should be aware of the possibility of false-positive microbiology results and consider contaminated products when unexplainable results are found. 

Appendix Additional information on *Bordetella parapertussis* pseudo-outbreak caused by contaminated swabs, the Netherlands.
