# Supplementary material for: Pseudo-Outbreak of Bordetella parapertussis Caused by Contaminated Swabs in the Netherlands
Source: Emerg Infect Dis. 2022 Apr;28(4):890–2. doi: 10.3201/eid2804.212097 (PMC8962902; doi:10.3201/eid2804.212097)
Supplement: Appendix — Additional information on Bordetella parapertussis pseudo-outbreak caused by contaminated swabs, the Netherlands. [file 21-2097-Techapp-s1.pdf]

Article DOI: https://doi.org/10.3201/eid2804.212097

Pseudo-Outbreak of *Bordetella parapertussis* Caused by Contaminated Swabs, the Netherlands

Appendix

Notifications

Notifications were sent by the Ministry of Health, the Netherlands, to members of Labinf@ct regarding nasopharyngeal swabs contaminated with *Bordetella parapertussis* insertion sequence 1001 (*IS1001*) DNA during 2020–2021. Notifications were provided in Dutch and English.

Rijksinstituut voor Volksgezondheid en Milieu
Ministerie van Volksgezondheid, Welzijn en Sport

Labinf@ct: B. parapertussis (2)

20 juli 2021

In dit bericht:

- Meerdere swabs betrokken bij fout-positief resultaat in een *B. parapertussis*-PCR: 8 lotnummers e-swabs (oranje dop) en 2 lotnummers droge swabs (blauwe dop)

Bericht:

In het Labinf@ct *B. parapertussis* van 3 juni 2021 werd melding gemaakt van een fout-positief signaal in de *B. parapertussis*-PCR bij gebruik van e-swabs. Wij deden in dat Labinf@ct een oproep aan de laboratoria om de bevindingen van het gebruik van deze swabs te delen.

De informatie die wij verkregen, en die werd gedeeld door de medisch moleculair microbiologen in de WMDI (werkgroep moleculaire diagnostiek van infectieziekten), leidde ertoe dat *IS1001*-positieve wattentips zijn gevonden bij Copan swabs zowel in afnamesets met een oranje dop als in droge swabs (blauwe dop) :

- E-swabs met oranje dop van Copan: Er zijn in totaal door 6 laboratoria 8 verschillende lotnummers gemeld met een fout-positieve reactie: lotnummers lot# 21087100; 203038000; 202323100; 202433300; 203193300; 201896300; 202011501; 2031933.

- (Droge) swab met blauwe dop van Copan: lot# 2004732; 202472700.

Het gaat bij de fout-positieve reactie om detectie van het *IS1001*-element; de Ct-waarden liggen boven de 34; het *IS1002*-element lijkt geen reactie te geven.

Er zijn door ten minste 3 laboratoria uitslagen als positief gerapporteerd aan de inzenders. Alleen als er sprake is van een bijpassend klinisch beeld is een positieve PCR-uitslag voor *B. parapertussis* meldingsplichtig. Ook als er sprake is van meerdere gevallen in een instelling kan er een meldingsplicht (artikel 26 Wet publieke gezondheid) bestaan. Voor de complete meldingscriteria, zie LCI-richtlijn Kinkhoest.

Een aantal gemelde casussen is op basis van fout-positiviteit herroepen. Bij een deel van de andere casussen is niet meer te achterhalen of de melding terecht was of niet. Deze zullen dan niet worden herroepen.

De leverancier (MLS) en producent (Copan) en de IGJ zijn geïnformeerd. Hoeveel patiënten ten onrechte zijn behandeld, is ter beoordeling aan de IGJ. De omvang van het probleem is niet vast te stellen aan de hand van de gemelde casussen, omdat een deel niet hoeft te zijn gemeld.

De leverancier MLS heeft inmiddels, namens Copan, een brief gestuurd naar de gebruikers waarin zij melden ook een positief resultaat te hebben gevonden in reeds onderzochte swabs

met oranje dop, waarbij wel/geen detectie afhankelijk is van het gebruikte platform. Daarin wordt vermeld:

Copan cannot exclude the presence of traces of nucleic acid materials into the eSwab that may be detected as Bordetella parapertussis under certain testing conditions.

As a good clinical practice, diagnostic companies and end users are required to verify the compatibility of the transport system with the specific molecular assay before use.

[…]

Copan became aware of this combination during the ongoing investigation.

Samenvattend zijn de wattenstokken gedurende het productieproces gesteriliseerd middels ioniserende straling. Zie ESwab-Package-Insert.pdf (copanusa.com). Kweken zijn negatief, maar DNA-resten zijn platform-afhankelijk aantoonbaar gebleven.

Contactgegevens

- **Overleg met uw regionale GGD** over casuïstiek over mogelijk verdachte gevallen in uw regio. Het nummer is te vinden op www.ggd.nl.
- **RIVM-LCI:** tel. 030-2747000 (ook buiten kantooruren bereikbaar)
- **RIVM dd. arts-microbioloog**: IDS-ddmicrobioloog@rivm.nl tel. 030-2743487 (ook buiten kantooruren bereikbaar)

Auteurs: Ellen Mascini, Guido Bastiaens, Jacky Flipse, Angelino Tromp vanuit Rijnstate; Erik van Hannen (WMDI); Erik Schaftenaar (AZN); Bettie Voordouw, Titia Kortbeek, George Sips, Michelle Stadlander, Dimphey van Meijeren, Hans van den Kerkhof (RIVM-CIb)

Bericht verstuurd aan: leden Labinf@ct

Labinf@ct: B. parapertussis (2)

July 20th 2021

This letter:

- Multiple batches of swabs involved in false-positive results for B. parapertussis-PCR: 8 batches e-swabs (orange cap) and 2 batches dry swabs (blue cap).

Notification:

In the Labinf@ct *B. parapertussis* of June 3rd 2021, we reported that the use of e-swabs can lead to false-positive *B. parapertussis*-PCRs. We requested laboratories to share their experiences and findings with us.

The information which we received, and which was shared by the Medical Molecular Microbiologists in the WMDI (working group Molecular Diagnostics in Infectious diseases), led to the identification of *IS1001*-positive swab tips in Copan swabs. These Copan swabs were found both with orange caps (sampling set) and with blue caps (dry swabs).

- E-swabs with orange cap: Six laboratories reported 8 different batch numbers with false-positive PCRs: lot# 21087100; 203038000; 202323100; 202433300; 203193300; 201896300; 202011501; 2031933.

- (dry) swabs with blue caps: lot# 2004732; 202472700.

All false-positive PCRs concern the detection of the IS1001-element; Ct values typically are Ct34 or higher. The IS1002-element has not been detected up to now.

At least three laboratories have reported positive results to physicians. Notification of *B. parapertussis*-PCRs are only obligatory in cases with pertussis-like symptoms. Additionally,

notifications may be obligatory if multiple cases are found within an institution (article 26, law on public health). For the full list of criteria, see the LCI guideline Pertussis.

Several of the notified cases have been retracted due to false-positive PCRs. In other cases, the possibility of a false-positive PCR cannot be investigated. Consequently, these notifications will not be retracted.

The distributor (MLS), producer (Copan) and the national health authority have been informed. The national health authority will investigate whether patients have incorrectly received treatment. The extent of the problem cannot be determined based on the notifications, as notifications are not mandatory for all positive PCRs.

All e-swab users have received a letter from the distributor MLS and Copan stipulating that Copan has confirmed the possibility of false-positive PCRs on swabs with an orange cap. Moreover, the false-positivity depends on the sensitivity of the used kit and platform. The letter further mentions:

Copan cannot exclude the presence of traces of nucleic acid materials into the eSwab that may be detected as Bordetella parapertussis under certain testing conditions.

As a good clinical practice, diagnostic companies and end users are required to verify the compatibility of the transport system with the specific molecular assay before use.

[…]

Copan became aware of this combination during the ongoing investigation.

As part of the production process, e-swabs are sterilised with ionizing radiation. See: ESwab-Package-Insert.pdf (copanusa.com). In line with this, all bacterial cultures are negative, however DNA remains detectable depending on the kit and platform used.

Authors: Ellen Mascini, Guido Bastiaens, Jacky Flipse, Angelino Tromp vanuit Rijnstate; Erik van Hannen (WMDI); Erik Schaftenaar (AZN); Bettie Voordouw, Titia Kortbeek, George Sips, Michelle Stadlander, Dimphey van Meijeren, Hans van den Kerkhof (RIVM-CIb)

Sent to: members of Labinf@ct

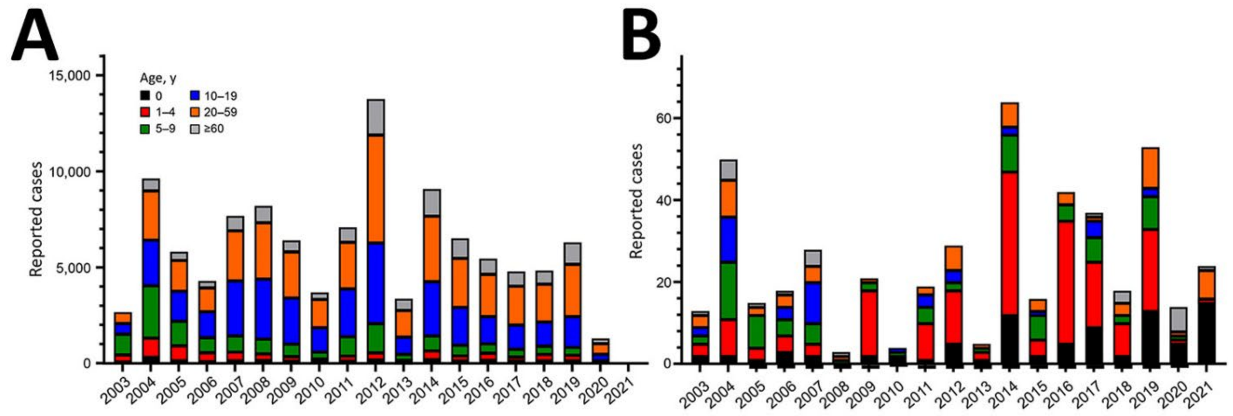

Appendix Figure. Comparison of notified cases of *Bordetella pertussis* and *B. parapertussis* registered by the National Institute for Public Health and the Environment (RIVM), the Netherlands, 2003–June 2021. A) *Bordetella pertussis*; B) *B. parapertussis*. Annual reported cases are stratified by age groups.
